# GANT-61 induces cell cycle resting and autophagy by down-regulating RNAP III signal pathway and tRNA-Gly-CCC synthesis to combate chondrosarcoma

**DOI:** 10.1038/s41419-023-05926-6

**Published:** 2023-07-24

**Authors:** Yifeng Sun, Qiongxuan Fang, Wei Liu, Yi Liu, Chunming Zhang

**Affiliations:** 1 Department of Orthopedic Surgery, The First Affiliated Hospital of Shandong First Medical University &Shandong Provincial Qianfoshan Hospital, Shandong Key Laboratory of Rheumatic Disease and Translational Medicine, Jinan, Shandong 250014 PR China; 2 Department of Surgery, Heidelberg University Hospital, Heidelberg University, Heidelberg, Germany; 3 Department of Surgery, Ulm University Hospital, Ulm University, Ulm, Germany; 4 MOE Key Laboratory of Cell Proliferation and Differentiation, School of Life Sciences, Peking University, Beijing, 100871 China

**Keywords:** Bone cancer, Macroautophagy

## Abstract

Chondrosarcoma is ineffective for conventional radiotherapy and chemotherapy with a poor prognosis. Hedgehog (Hh) signal pathway plays a crucial role in tumor growth and progression, which is constitutive activated in chondrosarcoma. GLI transcription factors as targets for new drugs or interference technology for the treatment of chondrosarcoma are of great significance. In this study, we indicated that the Hedgehog-GLI1 signal pathway is activated in chondrosarcoma, which further enhances the RNAP III signal pathway to mediate endogenous tRNA fragments synthesis. Downstream oncology functions of endogenous tRNA fragments, such as “cell cycle” and “death receptor binding”, are involved in malignant chondrosarcoma. The GANT-61, as an inhibitor of GLI1, could inhibit chondrosarcoma tumor growth effectively by inhibiting the RNAP III signal pathway and tRNA-Gly-CCC synthesis in vivo. Induced G2/M cell cycle resting, apoptosis, and autophagy were the main mechanisms for the inhibitory effect of GANT-61 on chondrosarcoma, which correspond with the above-described downstream oncology functions of endogenous tRNA fragments. We also identified the molecular mechanism by which GANT-61-induced autophagy is involved in ULK1 expression and MAPK signaling pathway. Thus, GANT-61 will be an ideal and promising strategy for combating chondrosarcoma.

## Introduction

Chondrosarcoma is the second most common primary bone malignancy, which is ineffective for conventional radiotherapy and chemotherapy with a poor prognosis. Surgical resection is currently the only treatment for chondrosarcoma. Therefore, there is an extremely urgent need to explore new therapeutic targets to combat this tumor [1].

Hedgehog (Hh) signal pathway plays a crucial role in tumor growth and development, which is constitutive activated in chondrosarcoma [2]. In mammals, the three GLI factors encode context-dependent activities with GLI1 being mostly an activator and GLI3 often a repressor, GLI2 may be an activator or repressor in different cells at different times [3]. GLI transcription factors as targets for new drugs or interference technology for the treatment of chondrosarcoma are of great significance [4, 5]. In our early study, we have shown that GLI1 inhibition could significantly inhibit the growth and development of chondrosarcoma in vitro, but more work still needs to be carried out to verify the effect of GLI1 inhibition and fully elucidate GLI1 functions in vivo. A promising therapeutic agent is GANT-61, which directly binds to the transcription factor GLI1/2, inhibits tumor cell proliferation and suppresses tumor formation in many preclinical studies [6]. Fu et al. showed that GANT-61 inhibits pancreatic cancer stem cell growth in vitro and NOD/SCID/IL2R gamma null mice xenograft [7]. Malin Wickström et al. proved that GANT-61 enhances the effects of chemotherapeutic drugs used in the treatment of neuroblastoma in an additive or synergistic manner and reduces the growth of established neuroblastoma xenografts in nude mice [8]. Therefore, Targeting the Hh pathway by using GANT-61 may provide a promising therapy for chondrosarcoma.

Up to now, some new perspectives and biology functions appeared against refractory malignant tumors, such as those below. (1) RNA polymerase III (RNAP III) is a nucleoprotein that transcribes DNA to synthesize tRNA. tRNA fragments (tRFs) have been shown to have crucial regulatory roles in cancer biology. However, the contributions of tRFs to chondrosarcoma remain largely unknown [9, 10]. (2) Autophagy has been reported to either promote or inhibit tumorigenesis, development, and chemotherapy resistance. It is worth exploring these mechanisms in chondrosarcoma, as they may reveal remarkable insights into novel means for regulating chondrosarcoma growth, recurrence, and metastasis [11, 12]. (3) Disruption of cell cycle checkpoints represent a series of tightly integrated events that allow the cell to grow and proliferate. Targeting cell-cycle checkpoints may provide substantial improvement to cancer therapy [13, 14]. In this study, we devoted ourselves to demonstrating the relationship among the Hh signal, RNAP III transcription pathway, and tRFs. We reported the antitumor activity of GANT-61 and its mechanisms (cell cycle arrest and autophagy) for chondrosarcomas in vivo. This report shows the possibility that GLI1 inhibitors might be a potential clinical compound in the treatment of chondrosarcomas.

## Results

### Activated Hedgehog-Gli1 signal pathway contributed to chondrosarcoma

**Fig. 1:**
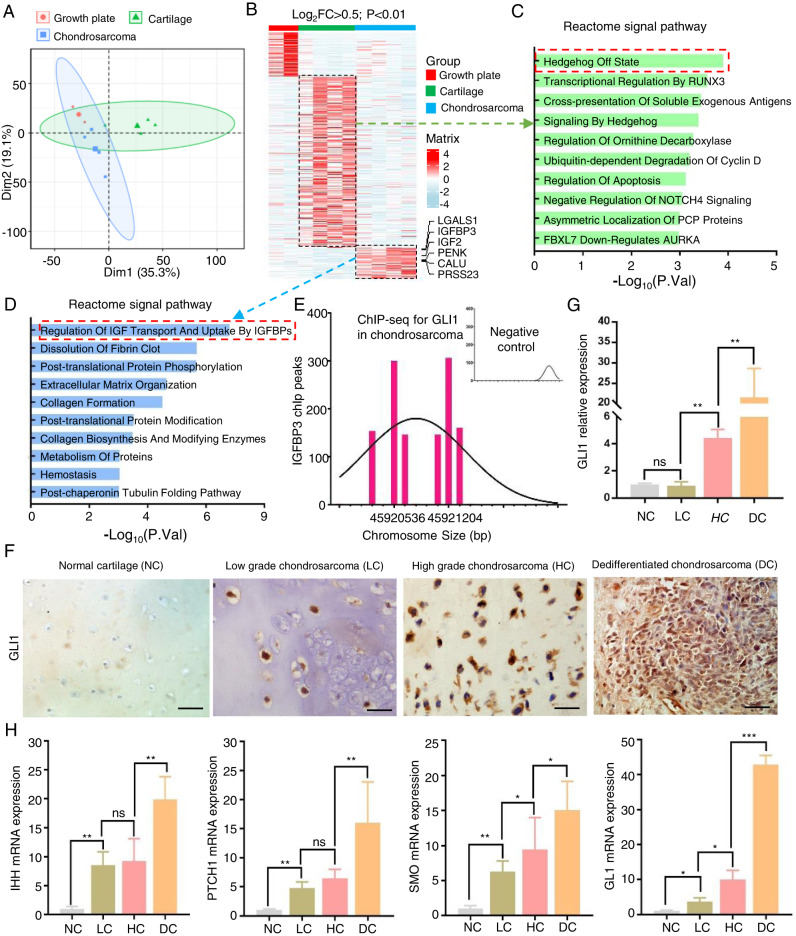
Activated Hedgehog-Gli1 signal pathway contributed to chondrosarcoma. **A** We processed the GSE30835 data from GEO datasets including 2 growth plates, 4 normal cartilages, and 4 chondrosarcomas. Principal component analysis (PCA) plot of transcriptional profiles with 95% confidence ellipsoids. **B** Then heat map showed the significantly different genes in these three groups (log2FC > 1; *P* < 0.01). **C**, **D** Enrichment of BioPlanet signal pathways in normal cartilage and chondrosarcoma group respectively. **E** ChIP-Seq was used to map IGFBP3 binding sites for GLI1 in chondrosarcoma. **F** Immunohistochemistry (IHC) analyses for GLI1 were performed in tissue sections from the normal articular cartilage (NC), low-grade (LC), high-grade (HC), and dedifferentiated chondrosarcoma (DC). **G** Densitometry of IHC were performed for quantification by using ImageJ 1.53 (**P < 0.01). **H** mRNA expression changes of IHH, PTCH1, SMO, and Gli1 were detected using qPCR in the NC, LC, HC, and DC groups (*P < 0.05, **P < 0.01).

To explore the potential activated signal pathways in chondrosarcoma, we processed the GSE30835 data from GEO datasets, which included two growth plates, four normal cartilages, and four chondrosarcomas, but excluded the Ollier disease [15]. After quality control, we performed a PCA analysis and found that the normal cartilage and chondrosarcoma have different developmental trajectories from the growth plate (Fig. 1A). Finally, we found 326 up-regulated genes in normal cartilage and 116 up-regulated genes in chondrosarcoma respectively (log_2_FC > 0.5, *p* value < 0.01, Fig. 1B). Of these, the Hh signal pathway is in off sate in normal cartilage, while “Regulation of IGF Transport And Uptake By IGFBPs” signal pathway is activated in chondrosarcoma (Fig. 1C, D). In the previous study, IGFBP3 is regulated by GLI signaling in the progression to malignant chondrosarcoma [16]. We also found that GLI1 could act directly on IGFBP3 to regulate the downstream signal in one chip-seq analysis (GSE100936) (Fig. 1E). GLI1 is the final and most crucial transcript factor in the hedgehog signal pathway [17]. Then, we collected 10 normal cartilages, 26 low-grade (I–II), 33 high-grade (III), as well as 25 dedifferentiated chondrosarcomas to detect the expression of GLI1, which has no significant difference between normal cartilage and low-grade chondrosarcoma, but obviously increased in high-grade and dedifferentiated chondrosarcoma in our IHC staining (Fig. 1F, G). Prehypertrophic and hypertrophic chondrocytes secrete IHH, which stimulates proliferation of growth plate chondrocytes. The Hedgehog (HH) pathway activates GLI-mediated transcription through transmembrane proteins, including patched 1 (PTCH1) and smoothened (SMO) [5]. Then, Using qRT-PCR, we measured the expression of IHH, PTCH1, SMO, and GLI1 and found that their levels were significantly lower in normal articular cartilage compared to any other chondrosarcomas, particularly in dedifferentiated chondrosarcoma (Fig. 1H). According to the results, we deeply deem that Hedgehog-GLI1 pathway is on state and further activates the downstream signals in chondrosarcoma.

### Hedgehog-GLI1 signal mediated the RNAP III signal pathway and tRNA synthesis to regulate cell cycle and death receptor binding in chondrosarcoma

**Fig. 2:**
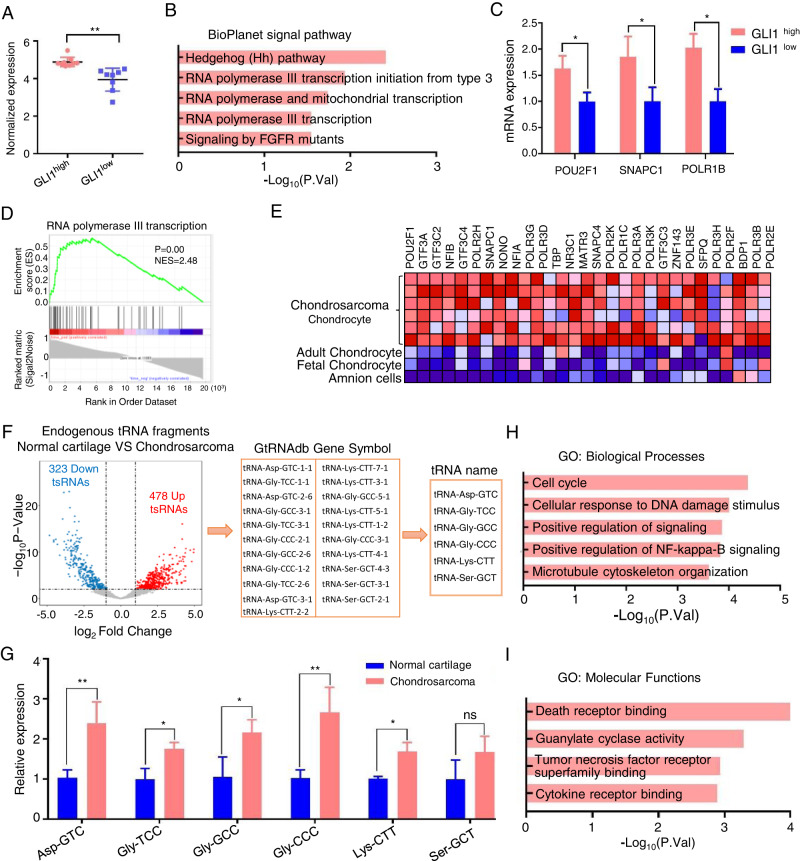
Hedgehog-GLI1 signal mediated the RNAP III signal pathway and tRNA synthesis to regulate cell cycle and death receptor binding in chondrosarcoma. **A** The expression of GLI1 was dichotomized using the median as a cutoff to define ‘high’’ or ‘low’’ expression categories for each sample obtained from GSE12475 (**P < 0.01). **B** Enrichment of BioPlanet signal pathways in GLI1^high^ group. **C** mRNA expression changes of POU2F1, SMAPC1, POLR1B were detected using qPCR in GLI1^high^ and GLI1^low^ groups (*P < 0.05). **D** The GSEA enrichment plot showed RNA polymerase III transcription pathway is enriched in the GLI1^high^ group (*P* < 0.01, NES = 2.48). **E** The heatmap representation of this subset of genes. **F** Volcano plot of significantly different endogenous tRNA fragments (tRFs) (|log2FC| > 1.0, *P < 0.05, **P < 0.01) between normal cartilage and chondrosarcoma, then we used the Genomic tRNA Database (GtRNAdb) to predict the tRNA gene. **G** Relative expression of tRNA-Asp-GTC, tRNA-Gly-TCC, tRNA-Gly-GCC, tRNA-Gly-CCC, tRNA-Lys-CTT, tRNA-Ser-GCT (*P < 0.05, **P < 0.01) between normal cartilage and chondrosarcoma. **H**, **I** Enrichment of GO Biological_Processes and Molecular Functions in tsRFuntion dataset (tsRFun).

To further determine the mechanism of the Hedgehog-GLI1 signal in malignant chondrosarcoma, we reanalyzed a gene expression profiling of 17 fresh frozen chondrosarcoma biopsies [18]. After normalizing the data, the expression of GLI1 was dichotomized using the median as a cutoff to define ‘high’’ or ‘low’’ expression categories for each sample (Fig. 2A). Furthermore, we compared the transcriptional profiles of GLI1^high^ to GLI1^low^ samples. A total of 542 differentially expressed transcripts (*p* < 0.05; |log_2_FC| > 0.25) were identified, and 117 of them were elevated in GLI1^high^ chondrosarcomas. We analyzed the BioPlanet signal pathways using the GLI1^high^ expressed transcripts to gain insight into the functional relevance. RNA polymerase III (RNAP III) transcription-relevant pathways were enriched, which are attributed to the Hedgehog-GLI1 pathway activation (Fig. 2B). We also validated these results using qRT-PCR to assess the level of GLI1 expression in our collected chondrosarcoma samples (excluding dedifferentiated chondrosarcoma to match the public microarray data). Our analysis identified 26 GLI1^low^ and 33 GLI1^high^ chondrosarcoma samples, which correlated with tumor grade (as shown in Fig. 1H) and suggests that GLI1 expression promotes chondrosarcoma progression. Notably, the hub genes of the RNAP III signaling pathway, such as POU2F1, SNAPC1, and POLR1B, were significantly higher in the GLI1^high^ group compared to the GLI1^low^ group (Fig. 2C). In addition, we processed chondrocyte-specific transcription factors that could directly convert human amnion cells into chondrosarcoma to demonstrate the RNAP III transcription signal pathway in chondrosarcoma progression. Enrichment plot GSEA indicated that RNAP III transcription signal pathway enriched in chondrosarcoma (*P* < 0.01, NES = 2.48) (Fig. 2D). The heatmap representation of this subset of genes was shown in Fig. 2E.

The abundant tRNAs in eukaryote cells that are synthesized by RNAP III have key functional roles [19]. Therefore, we analyzed a modulated expression of endogenous tRNA fragments (tRFs) in six normal cartilage and fourteen chondrosarcomas. We detected 478 up-regulated and 321 down-regulated tRFs in chondrosarcoma (|log_2_FC| > 1.0, *p* < 0.01, Fig. 2F). Then we used the Genomic tRNA Database (GtRNAdb) to predict the tRNA gene. Out of 21 enriched tRFs in the human species present in GtRNAdb, six tRNAs were detected. We confirmed the expression levels of these tRNAs using qPCR, which revealed that, except for Ser-GCT, the other tRNAs (tRNA-Asp-GTC, tRNA-Gly-TCC, tRNA-Gly-GCC, tRNA-Gly-CCC, and tRNA-Lys-CTT) showed significantly higher expression in chondrosarcoma compared to normal cartilage (Fig. 2G). Another dataset called tsRFuntion (tsRFun) was applied to predict the functions of tRFs target genes by GO enrichment analysis. The enrichment analysis highlighted “cell cycle” and “death receptor binding” were the most significant biological processes (BP) and molecular functions (MF) respectively (Fig. 2H, I). Taking all the results together, the Hedgehog-GLI1 signal mediated the RNAP III signal pathway and tRNA synthesis to regulate the cell cycle and death receptor binding in chondrosarcoma.

### GLI1 inhibitor (GANT-61) suppressed chondrosarcoma by inhibiting the RNAP III signal pathway and tRNA-Gly-CCC synthesis in vivo

**Fig. 3:**
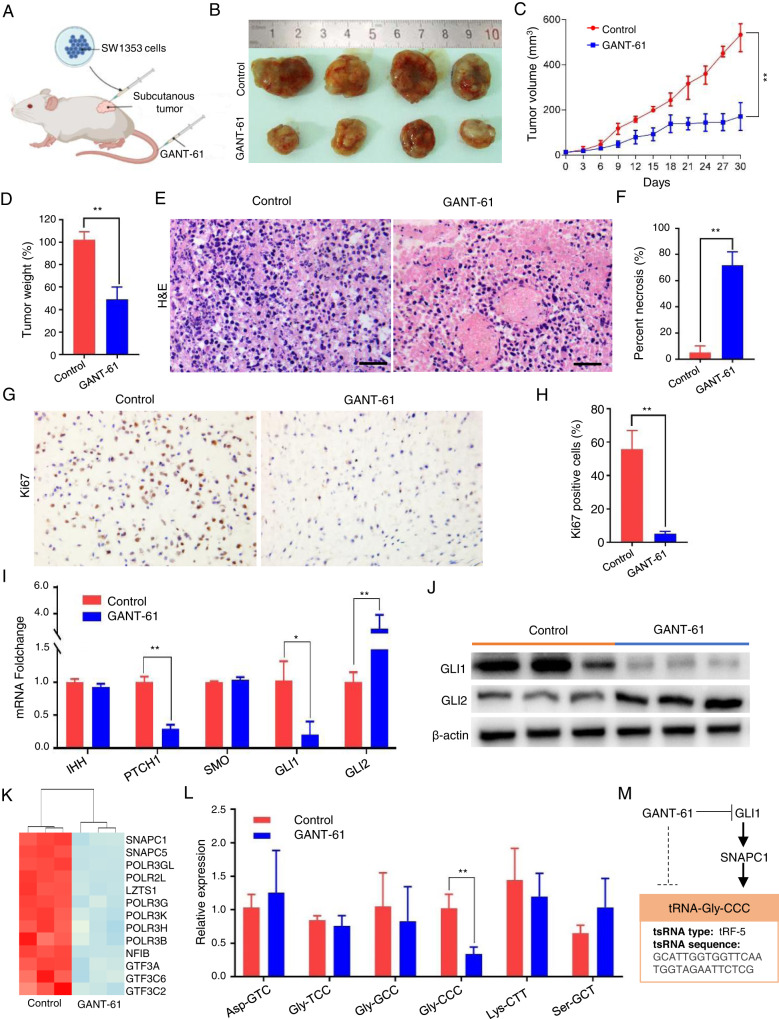
GLI1 inhibitor (GANT-61) suppressed chondrosarcoma by inhibiting the RNAP III signal pathway and tRNA-Gly-CCC synthesis in vivo. **A** Establishment of an in vivo xenograft mouse model. **B** Representative images of xenograft tumors. **C** Mice were treated with or without GANT-61 intravenous injection per three days for six times. Tumor volume growth curve was measured per three days for one month (**P < 0.01). **D** Tumor weights of mice from different treatment regimens (**P < 0.01). **E** Tumor tissue necrosis with or without GANT-61 detected by H&E staining. Five representative fields at a 400-fold magnification were counted per animal. **F** The mean necrosis rate between GANT-61 treated animals and the control group (**P < 0.01). **G** Tissue sections from GANT-61 treated animals and the control group were subjected to IHC analysis for Ki-67. **H** The number of Ki-67 positive cells was compared between GANT-61 treated animals and the control group (**P < 0.01). **I** mRNA expression changes of IHH, PTCH1, SMO, Gli1, and Gli2 were detected using qPCR in the two groups (*P < 0.05, **P < 0.01). **J** Western blot (WB) analysis was performed to assess the protein expression levels of Gli1, and Gli2. **K** Heat map of hub genes of RNAP III transcription signal pathway. **L** Relative expression of tRNA-Asp-GTC, tRNA-Gly-TCC, tRNA-Gly-GCC, tRNA-Gly-CCC, tRNA-Lys-CTT, tRNA-Ser-GCT (*P < 0.05, **P < 0.01). **M** Illustration of the relationship among Hedgehog-GLI1 signal, RNAP III transcription signal pathway, and tRNA-Gly-TCC.

As the Hedgehog-GLI1 signal pathway is activated in chondrosarcoma, we established a vivo subcutaneous xenograft model by SW1353 cells. After five days of xenograft, mice were treated with GANT-61 intravenous injection per three days for one month (Fig. 3A). Tumor volumes were calculated in the GANT-61 group were decreased by 53 ± 8.9%, as well as had a lower proliferation rate than the control group (Fig. 3B, C, *P* < 0.01). The tumor weights were also decreased by 51.2 ± 11.0% (Fig. 3D). The significant increase in regions of necrosis elicited by GANT-61 was assessed by microscopic examination of H&E-staining is shown in Fig. 3E, in which necrosis rate already accounted for ~70% when compared to 5% of the control group (Fig. 3F). Then we conducted an IHC test to detect tumor cell proliferation by examining the expression of Ki-67, and our findings indicate that GANT-61 significantly inhibits the number of Ki67-positive cells (Fig. 3G, H). To further test the functions of GANT-61 on Hh signal pathway, we assessed the key members IHH, PTCH1, SMO, GLI1, GLI2 in the hedgehog pathway by using qRT-PCR. We found that the PTCH1 and GLI1 expression was significantly decreased, while the GLI2 expression was increased (Fig. 3I). We assessed the protein levels of GLI1 and GLI2 and found consistent results with the mRNA levels, suggesting that GANT-61 primarily reduces GLI1 expression rather than GLI2 in chondrosarcoma (Fig. 3J). To validate whether the anti-tumor effects of GANT-61 were reflected at gene expression levels, we performed microarray-based transcriptomic profiling of chondrosarcoma subcutaneous tumor tissues. It was found that massive genes in vivo xenograft models were differentially expressed (|log_2_FC| ≥ 1; *p* < 0.01) following treatment with GANT-61. There were 1772 genes with up-regulated and 2479 genes with downregulated mRNA expression levels (supplement table 2). Following the analysis of the microarray data, the hub genes of the RNAP III signal pathway were downregulated (Fig. 3K). To test whether the downregulated RNAP III signal pathway affects the tRNA synthesis or not, the six upregulated candidate tRNAs in Fig. 3L were further checked by qRT-PCR, Interestingly, only tRNA-Gly-CCC was significantly declined in our study. SNAPC1 in the RNAP III signal pathway is the bridge genes between GANT-61 and tRNA-Gly-CCC, and the target sequence of tRNA-Gly-CCC was described in the Fig. 3M. All these findings imply that GANT-61 tends to inhibit chondrosarcoma tumor formation by suppressing the RNAP III signal pathway regulated tRNA-Gly-CCC expression in vivo.

### GANT-61 blocks the chondrosarcoma cells in G2/M cell cycle phase

**Fig. 4:**
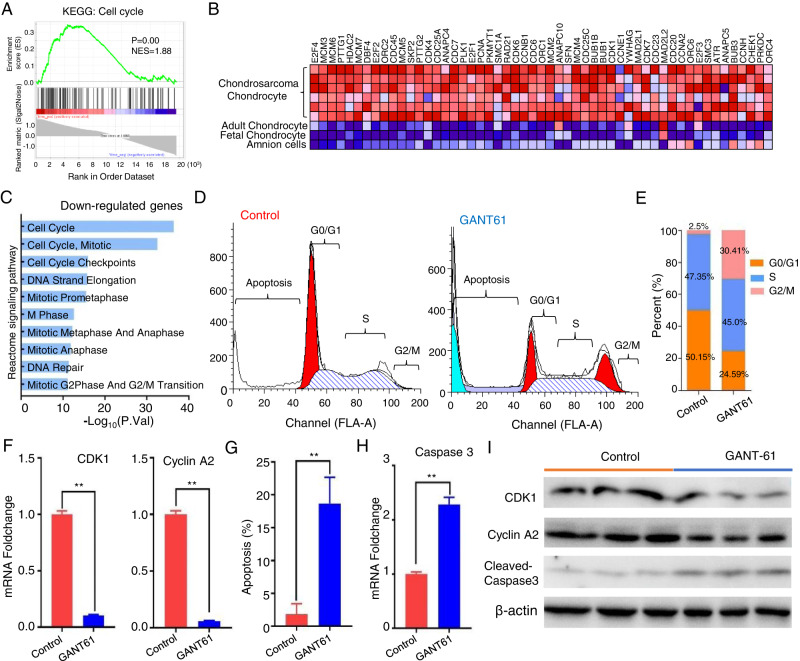
GANT-61 blocks the chondrosarcoma cells in the G2/M cell cycle phase. **A** The GSEA enrichment plot showed cell cycle pathway is enriched in the chondrosarcoma (*P* < 0.01, NES = 1.88). **B** The heatmap representation of this subset of genes. **C** BioPlanet signal pathway enrichment analysis by using the downregulated genes in GANT-61 treatment. **D** Cell cycle and apoptosis analysis stained by propidium iodide in the isolated tumor cells of the two groups with flow cytometry. **E** The bar chart shows the percentage of cells in the cell cycle. **F** qPCR analysis for CDK1 and Cyclin A2 (**P < 0.01). **G** The bar graph showed a significant increase in apoptosis rate in GANT-61 treatment group (**P < 0.01). **H** qPCR analysis for Caspase3 (**P < 0.01). **I** Protein expression levels of CDK1, Cyclin A2, and cleaved-Caspase-3 were assessed using Western blot analysis.

Just as described in Fig. 2H, the cell cycle signal pathway is activated in chondrosarcoma through the endogenous tRNA fragments enrichment analysis. We also acquired a consistent result in transcriptomic expression levels, which was that cell cycle pathway is significantly elevated in chondrosarcoma when compared to the amnion cells, fetal and adult chondrocytes by GSEA analysis (*P* < 0.01, NES = 1.88) (Fig. 4A, B). We carried out a pathway enrichment analysis by using the down-regulated genes, and found that the cell cycle signal pathway was successfully suppressed by the GANT-61 in our microarray data (Fig. 4C). We further confirmed that GANT-61 treatment caused marked accumulation of a G2/M population in the isolated tumor cells of the animal models with flow cytometry (Fig. 4D). G2/M arrest population was 30.41 ± 4.67% in those animals exposed to GANT-61, while approximately 2.5 ± 0.53% of the control group (Fig. 4E). The underlying mechanism behind the inhibition of the G2/M checkpoint by GANT-61 can be attributed to its ability to impede the molecular changes of CDK1 and Cyclin 2A at both the mRNA and protein levels. (Fig. 4F, I).

### GANT-61 induced chondrosarcoma cell death through apoptosis and autophagy

**Fig. 5:**
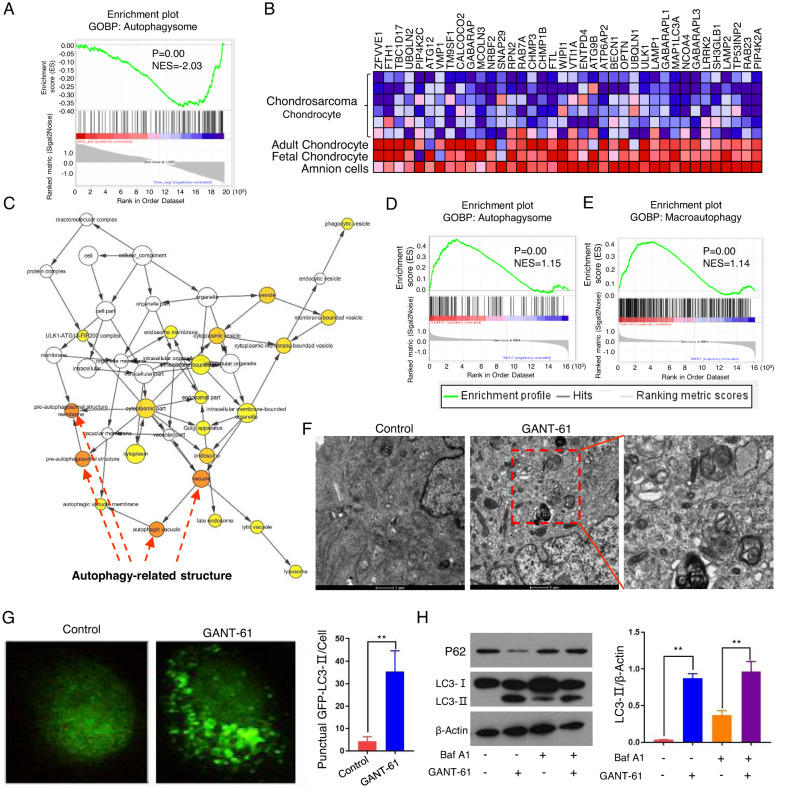
GANT-61 induced chondrosarcoma cell death through apoptosis and autophagy. **A** The GSEA enrichment plot showed autophagysome is decreased in the chondrosarcoma (*P* < 0.01, NES = −2.03). **B** The heatmap representation of this subset of genes. **C** The gene-concept network analysis was performed and visualized in Cytoscape. **D**, **E** GSEA analysis of autophagysome (*p* < 0.01, NES = 1.15) and macroautophagy (*P* < 0.01, NES = 1.14) between GANT-61 and the control group. **F** Representative autophagosome-like vacuoles with double-membrane structures depict ultrastructures of autophagy. **G** Immunofluorescence analysis for LC3-II puncta in the isolated tumor cells of the two groups (left); Quantification of the number of LC3-II puncta (right, *P < 0.05, **P < 0.01). **H** Western blot was used to detect the expression of LC3B-II (an autophagosome marker) and P62 (a known autophagy substrate) with or without Baf A1 (left); Densitometry was performed for quantification, and the ratios of LC3B-II and P62 to b-actin are presented (right, **P < 0.01).

**Fig. 6:**
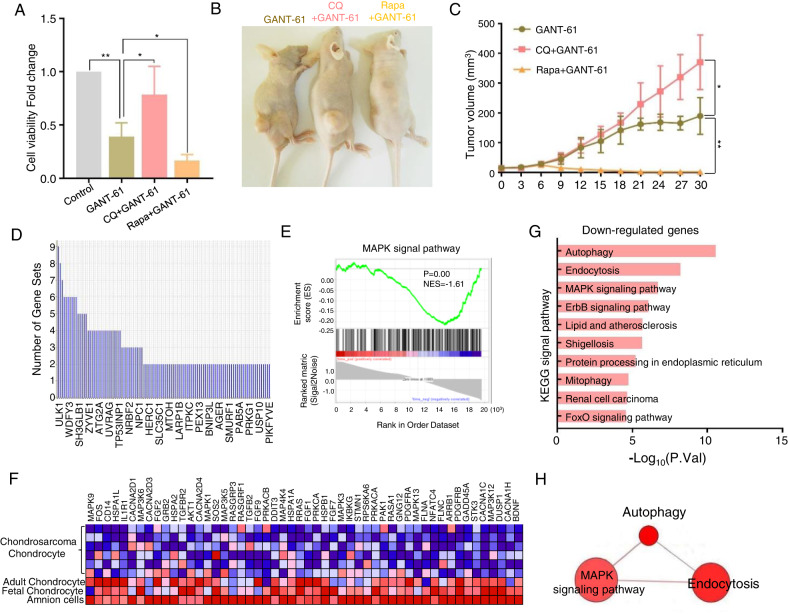
GANT-61 activated ULK1 and MAPK signaling pathway to regulate autophagy. **A** Isolated tumor cells were exposed to CQ (10 µM) or Rapa (100 nM) for 48 h. Then cell viability was tested using a CCK-8 assay (*P < 0.05, **P < 0.01). **B** Representative images of xenograft tumors in the GANT-61, GANT61 + CQ, and GANT61+Rapa groups. **C** Tumor volume growth curves were plotted per three days for one month for the above groups. (*P < 0.05, **P < 0.01). **D** Leading edge analysis to determine the most critical gene in autophagy. **E** The GSEA enrichment plot showed MAPK signal pathway is decreased in the chondrosarcoma (*P* < 0.01, NES = −1.61). **F** The heatmap representation of this subset of genes. **G** KEGG signal pathway enrichment analysis by using the upregulated genes in GANT-61 treatment. **H** The connections of autophagy and MAPK signal pathways in ConsensusPathDB datasets.

Given that the “Death receptor binding” function was observed in the endogenous tRNA fragments enrichment analysis of chondrosarcoma (Fig. 2I). Then we put our efforts into which cell death manners can be induced by GANT-61. Firstly, apoptosis rate distribution was determined by flow cytometry and increased by 16.89 ± 2.475% in GANT-61 treated group (Fig. 4D, G). And our findings suggest that the observed apoptosis is mediated by caspase-3 (Fig. 4H, I). Subsequently, we applied GSEA analysis to observe the autophagy level in chondrosarcoma. The interesting fact is that the autophagy in chondrosarcoma is at a lower level than in amnion cells, fetal and adult chondrocytes (*p* < 0.01, NES = −2.03) (Fig. 5A, B). In our transcriptomic expression data, the enrichment degrees of the cellular components also showed that differentially encoded product proteins were mainly distributed on vacuole, pre-autophagysomal structure membrane, autophagic vacuole by using the up-regulated genes in GANT-61 group (Fig. 5C). Meanwhile, GSEA analysis showed differentially up-regulated genes were related to the activation of autophagosome (*p* < 0.01, NES = 1.15) and macroautophagy (*P* < 0.01, NES = 1.14) (Fig. 5D, E). The KEGG signaling pathways analysis results also expounded that their encoding proteins were most involved in the autophagy signaling pathway (Fig. 6G).

To verify whether GANT-61 is involved in autophagy, TEM was used to detect the ultrastructures of autophagy. We found some representative features in the group of GANT-61 treatment, such as autophagic vacuole and autophagosome (Fig. 5F). Endogenous LC3-II visualized by immunofluorescence is recognized as an increased autophagosomal formation in living cells. We observed that GANT-61 significantly increased endogenous LC3-II puncta in the isolated tumor cells of the xenograft models (Fig. 5G). Detecting LC3-II and p62 combined with bafilomycin A1 (Baf A1, inhibitor of lysosomal degradation) by immunoblotting has become a reliable method for monitoring autophagy and autophagy-related processes. Our results show that GANT-61 increased the LC3-II localization in the cytoplasm and resulted in the process of transferring LC3-I to LC3-II with or without Baf A1 (Fig. 5H). A link between autophagy and cell death has been demonstrated using pharmacological (e.g., Rapamycin (Rapa) is a potent inducer of autophagy, chloroquine (CQ) is a lysosomotropic agent that has been suggested to inhibit autophagy) [20]. The autophagy inhibitor CQ (10 µM) significantly enhanced the viability of cells, but the autophagy inducer Rapa (100 nM) further depresses the viability in response to GANT-61 (Fig. 6A). In the xenograft model, the findings revealed that the GANT-61 + CQ group displayed a significant 61.3 ± 13.7% increase in tumor volume and a higher proliferation rate when compared to the GANT-61 group. Conversely, no tumor formation was observed in the GANT-61+Rapa group (Fig. 6B, C, *P < 0.01). Overall, the enrichment analysis of the up-regulated genes, along with the TEM, immunofluorescence technique, and immunoblotting results indicated that autophagy is silent in chondrosarcoma, but GANT-61 can arouse the autophagy to against this malignant sarcoma. Meanwhile, a combined strategy using GANT-61 and autophagy promoters, such as rapamycin, produces a superior antitumor effect.

### GANT-61 activated ULK1 and MAPK Signaling pathway to regulate autophagy

To declare the underlying mechanism of autophagy, GSEA analysis was also dedicated to performing leading edge analysis of the differential genes which was the most critical one. Genes in the subset list showed that ULK1 (unc-51 like autophagy activating kinase 1) had the highest impact on the biological process of autophagy (Fig. 6D). We uncovered that MAPK signal pathway is involved in the regulation of autophagy in our previous study [21]. Subsequently, we applied GSEA analysis to observe the MAPK signal level in chondrosarcoma and found that the MAPK signal is not in an activated state in chondrosarcoma (*p* < 0.01, NES = −1.61) (Fig. 6E, F). However, the autophagy and MAPK signal pathway were activated simultaneously when exposed to GANT-61 in the KEGG signal pathway analysis (Fig. 6G). Also, there is a potential correlation between autophagy and MAPK signaling pathway (Fig. 6H).

## Discussion

According to our previous research and the public data, we found that the Hedgehog-GLI1 signal pathway is aberrantly activated in chondrosarcoma. The GLI1 transcription factor mediated RNAP III transcription signal pathway and tRFs synthesis has crucial roles in regulating chondrosarcoma proliferation, cell cycle, and cell death. We demonstrated that GANT-61 induced cell cycle resting and autophagy by down-regulating RNAP III signal pathway and tRNA-Gly-CCC synthesis to suppress chondrosarcoma tumor growth in vivo. We also found that the MAPK signaling pathway played a significant role in the process of GANT-61 induced autophagy, ULK1 was also involved in the process. Therefore, we deeply believed that GANT-61 will be a novel approach for anticancer therapy for chondrosarcoma.

The Hh signal pathway is activated in various kinds of cancers [22]. Many studies showed that inhibition of GLI function holds strong potential to become a novel, clinically effective approach to treat malignant sarcoma. In recent years, the small molecule inhibitor GANT-61 is the most widely appreciated drug target of the GLI1/2, with improved potency and chemical stability, has strong and extensive anti-tumor effects in pancreatic cancer, breast cancer, rhabdomyosarcoma, glioblastoma, myeloid leukemia, osteosarcoma, melanomas, and so on [23, 24]. GANT-61 killed the sensitive tumor cells through anti-proliferation, oxidative stress, apoptosis, and autophagy, DNA damage, modulate the radiosensitivity and migration of cancer cells [25–28]. But the cancer therapy effect of GANT-61 on chondrosarcoma has not been reported. Possible mechanisms and implications of GANT-61 for clinical practice remain to be further studied in chondrosarcoma.

In parallel with the activated hedgehog signal pathway, our study highlighted that RNAP III transcription is more frequent in the GLI1 high-expression population in chondrosarcoma. RNAP III uniquely synthesizes and modifies most of the tRNAs that enhance mRNA decoding, which is primarily tied to the regulation of cell growth, cell cycle, and cell survival [29]. Notably, tRNA and tRFs have been reported to have crucial regulatory roles in a wide range of tumor biological processes [30, 31]. Goodarzi et al. reported that tRFs derived from tRNA-Asp, tRNA-Glu, tRNA-Tyr, and tRNA-Gly maintain cell stability with stress and increase cell proliferation in breast cancer [32]. Shao et al. covered that tRF-Leu−CAG promoted cell cycle progression and cell proliferation in lung cancer [33]. Some studies also found that silencing the expression of tRFs inhibited DNA synthesis, arrested cells at the G2/M phase, and decreased cell viability in common cancers, including lung cancer, colorectal cancer, prostate cancer, and breast cancer [34]. In our study, up-regulated tRFs was associated with the cell cycle and death-related molecular functions. GANT-61 is a useful drug that inhibited the RNAP III transcription pathway as well as tRNA-Gly-CCC synthesis, and further suppressed tumor growth by inducing G2/M cell cycle phase resting and programming cell death: autophagy and apoptosis.

Autophagy in most contexts facilitates tumorigenesis, and autophagy inhibition usually is an effective therapeutic strategy in many aggressive cancers [35]. In contrast to protective autophagy, induction of autophagic cell death has also been proposed as a possible tumor suppressor mechanism [36]. Our findings were expected to reflect the latest research that the status of autophagy is a key factor that determines the therapeutic response to Hh-targeted therapies. Firstly, autophagy is off sate in chondrosarcoma when compare to amnion cells, fetal and adult chondrocytes. Furthermore, GANT-61 induced autophagy promotes cell death against chondrosarcoma. Indeed, GANT-61 has been verified to induce tumor-suppressing autophagic cell death in pancreatic ductal adenocarcinoma (PDAC) and hepatocellular carcinoma (HCC), which has been used to monitor the relationship between specific GLI targeting and autophagy [17, 37, 38].

However, it is important to acknowledge that the concentration of GANT-61 used in this study is exceptionally high, and its safety needs to be thoroughly evaluated before proceeding to clinical trials. Consequently, our focus is to explore a novel delivery system that can effectively administer functional GANT-61 to chondrosarcoma-afflicted animals, thereby reducing its concentration while enhancing safety and efficacy in future investigations. Furthermore, in our study, GANT-61 reduced GLI1 expression while having no effect on GLI2. Given the overlapping and distinct activities of GLI proteins, including the fact that Gli1 acts as a transcriptional activator while Gli2 displays both activator and repressor functions, there is a need for further investigation of their functions.

**Fig. 7:**
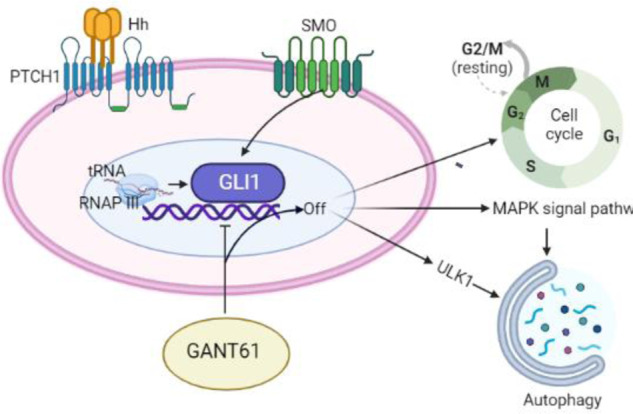
Overview of the signal pathways and biological functions in chondrosarcoma. The activated Hedgehog-Gli1 signal pathway in chondrosarcoma enhances RNAP III signaling for endogenoust RNA fragments synthesis. GANT-61 effectively inhibits tumor growth by targeting RNAP III and tRNA-Gly-CCC synthesis, inducing G2/M cell cycle arrest, apoptosis, and autophagy. GANT-61-induced autophagy involves ULK1 expression and MAPK signaling.

In conclusion, our results indicated that the Hedgehog-Gli1 signal pathway was activated in chondrosarcoma, which further enhanced the RNAP III signal pathway to mediate endogenous tRNA fragments synthesis. Downstream oncology functions of endogenous tRNA fragments, such as “cell cycle” and “death receptor binding”, were involved in malignant chondrosarcoma. The GANT-61 could inhibit chondrosarcoma tumor growth effectively by inhibiting the RNAP III signal pathway and tRNA-Gly-CCC synthesis in vivo. Induced G2/M cell cycle resting, apoptosis, and autophagy are the main mechanisms for the inhibitory effect of GANT-61 on chondrosarcoma, which correspond with the above-described downstream oncology functions of endogenous tRNA fragments. We also identified the molecular mechanism by which GANT-61-induced autophagy was involved in ULK1 expression and MAPK signaling pathway (Fig. 7). Thus, GANT-61 will be an ideal strategy for combating chondrosarcoma.

## Materials and methods

### Cell lines and reagents

The chondrosarcoma cell line used in the study is SW1353 (ATCC HTB-94; Manassas, VA, USA), which was maintained in L-15 medium with 10% FBS and 1% antibiotics. The reagent GANT-61 (S8075), Chloroquine (S6999), Bafilomycin A1 (S1413), and Rapamycin (S1039) were purchased from Selleckchem. Polyclonal antibodies against P62 (P0067), and LC3B (L7543) were obtained from Sigma-Aldrich. Anti-GLI1 (2643), Anti-GLI2 (18773), Anti-β-actin (4970), Anti-Ki-67 (34330), Anti-CDK1 (77055), Anti-Cyclin A2 (67955), Anti-Cleaved-Caspase3 (9664) were purchased from Cell Signaling Technology.

### Human articular cartilage and chondrosarcoma specimens

10 normal cartilages, 26 low-grade (I–II), 33 high-grade (III), as well as 25 dedifferentiated chondrosarcomas specimens were obtained in accordance with the approved protocols of the Institutional Ethics Review Boards of The First Affiliated Hospital of Shandong First Medical University &Shandong Provincial Qianfoshan Hospital. Informed written consent was obtained from all patients for the experimental use of surgical specimens, following the hospital’s ethical guidelines. Excisions obtained after surgery were appropriately preserved for the experiment.

### Xenograft animal model and primary cells isolation

Six weeks old BALB/c female nude mice (Charles River, Beijing, China) were inoculated subcutaneously in the right flank with SW1353 cells (2 × 10^6^ in 100 μL PBS). Firstly, eight mice were randomly divided equally into two groups. 100 μL GANT-61 (60 mg/kg) or ethanol (negative control) were injected intravenous every 3 days a total of 6 times beginning at day 5 after tumor inoculation. Secondly, fifteen mice were randomly divided into three groups, with equal numbers in each group. They were intravenously injected with GANT-61 (60 mg/kg) in combination with the autophagy inhibitor CQ (30 mg/kg), or the autophagy promoter Rapa (0.20 mg/kg), every 3 days, for a total of 6 injections, starting from day 5 after tumor inoculation, to assess their effects on tumor progression. After injection, mice were investigated, and tumor volumes were calculated every 3 days using a caliper (tumor volume = (length × width^2^)/2). All tumor tissues were harvested 30 days after treatment. Tumor samples were processed for gene expression microarray analyses, qPCR analysis, HE staining, and transmission electron microscopy (TEM). Primary cells were isolated from the tissue according to published techniques [39]. Briefly, Chop these pieces with a surgical blade cutting object, then add 5 ml trypsin-EDTA solution, and incubate for 10–20 min at 37 °C. Repeat spin and filtered 2 ~ 3 times, resuspend the cells with medium, and aliquot into a 6-well plate for the flow cytometry and immunofluorescence analysis.

### Microarray processing

Total RNA from tumor tissues was extracted using a Total RNA Miniprep Kit (T2010S, NEB). The quality of the isolated RNA was estimated using a NanoDrop 2000 (Thermo, China) and Agilent 2100 (Agilent, China). Triplicate samples for each group with A260/A280 ratio within 1.8–2.1 between 1.8 and 2.1 and a 28S/18S ratio between 1.5 and 2 were further processed for gene expression microarray analyses according to the manufacturer protocol (PrimeView Human Gene Expression Array, China). In this process, summary statistics were computed for each array and then compared across the arrays.

### Microarray data and public data analysis

Analyses were carried out with software package R (version 4.1.2) as previously described [40]. Significance analysis was performed by using the “limma” package. Principal component analysis was performed to evaluate the availability and quality of gene expression microarray. *P* value and absolute fold change (|Fold Change|) between the two experimental conditions (GANT-61 vs. Control) were used to identify the statistically most significant changes in gene expression. Gene Set Enrichment Analysis (GSEA) was used to explore the underlying mechanisms by identifying a priori-defined set of genes that shows statistically significant, concordant differences between two biological states. The gene-concept network analysis was performed by using the R packages ‘clusterProfiler’ and visualized in Cytoscape.

Total five human datasets (GSE30835, GSE100936, GSE12475, GSE29745, GSE86576) in GEO (https://www.ncbi.nlm.nih.gov/geo/) were collected [15, 18, 41, 42]. And the necessary documents were also respectively downloaded to annotate the respective probe sets into gene symbol sets. Enrichr (https://maayanlab.cloud/Enrichr/) is a robust web-server that contains many types of datasets, among which we used the Reactome signal pathway database, BioPlanet signal pathway database, KEGG pathway database, GO Biological_Processes, GO Molecular Functions. ChIP-seq data were analyzed by IGV_2.15.2 software. GtRNAdb (http://gtrnadb.ucsc.edu/) and tsRFun (http://rna.sysu.edu.cn/tsRFun/index.php) datasets were used to analyze the endogenous tRNA fragments.

### Cell viability assay

Tumor cell viability was tested using cell counting kit-8 (CCK8) assay. Firstly, 5000 cells/well were suspended in 200 μL of medium and incubated overnight in 96-well plates. After 48 h exposure to different treatment, then add 10 μL of CCK8 solution to each well and incubate for 2–4 h. Absorbance at 450 nm was observed using a microplate reader.

### RNA extraction and quantitative PCR analysis

Total RNA from tumor tissues was extracted using the Total RNA Miniprep Kit (T2010S, NEB). Then the RNA was reverse transcribed into cDNA in a reverse transcription reaction with RevertAid H Minus First Strand cDNA Synthesis Kit (k1632, Thermo Fisher). qPCR can be carried out sequentially in the same tube using a two-step qPCR Kit (M3003E, NEB) with LightCycler 480II (Roche; Basel, Switzerland). The primers used for amplification of IHH, PTCH1, SMO, GLI1, GLI2, CDK1, Cyclin A2, and Caspase 3 transcripts were shown in Supplement Table 1. The tRNA primer sets perform superbly with the rtStar™ tRNA Pretreatment & First-Strand cDNA Synthesis Kit (CAT# AS-FS-004) and Arraystar SYBR Green qPCR Master Mix.

### Transmission electron microscopy

Fresh tumor tissues were cut to 5 mm × 5 mm size, then 3% glutaraldehyde was added for 3 h at 4 °C for fixation. Ultrathin sections (100 nm) were prepared, stained with uranyl acetate and lead citrate, and examined under Transmission Electron Microscopy (TEM) (H-600; Hitachi, Tokyo, Japan).

### Western blotting and Immunofluorescence analysis

Equal amounts of proteins were collected from different tissue lysates of the two groups, and standard procedures of Western blotting were performed as described previously [21]. Primary cells were isolated for immunofluorescence observation, incubated with rabbit polyclonal anti-LC3B Ab (1:200) at 4 °C overnight, and then reacted with anti-rabbit IgG conjugated with Dylight 488 (1:400) at room temperature for 2 h. After washing with PBS, the puncta LC3B of cells were mounted on a vectashield and visualized using high-resolution microscopy (ZEISS ZEN Microscopy).

### H&E staining and Immunohistochemistry (IHC)

Harvested tissues were embedded in paraffin, Paraffin sections were subjected to Hematoxylin and Eosin (H&E) Staining and reacted with primary antibody according to manufacturer’s specification. For IHC, HRP-labeled polymer secondary antibody was applied. Five representative fields at a 400-fold magnification were counted per animal. Densitometry of IHC was performed for quantification by using ImageJ1.53.

### Cell cycle and apoptosis analysis by flow cytometry

Isolated tumor cells were fixed in 70% ethanol, and permeabilized in Triton X-100. Dispersed into single cells with RNAse A, and stained with Propidium Iodide kit (P1304MP, Thermo Fisher) according to the manufacturer’s instructions and analyzed by flow cytometry.

### Statistical analysis

The R software (version 4.1.2) and GraphPad Prism 7.0 statistical software were used to perform bioinformatic analysis and statistical analyses. Data were analyzed using one-way analysis of variance (ANOVA) with the Bonferroni multiple comparison test. Unpaired t-tests were employed for comparing between two groups. The data are presented as mean ± S.D. Statistical significance was defined as *P* < 0.05.

## Supplementary information

Supplementary table 1. Human primers sequences.

Supplementary table 2. Significant different genes between GANT-61 treatment animal and the control group.

Full western blot

## Data Availability

The accession number for the microarray data reported in this paper is OMIX002572-01. These data have been deposited in the Genome Sequence Archive under project PRJCA013766.

## Code availability

The code used and/or analyzed during the current study are available from the corresponding author on reasonable request.
