# Supplementary material for: GANT-61 induces cell cycle resting and autophagy by down-regulating RNAP III signal pathway and tRNA-Gly-CCC synthesis to combate chondrosarcoma
Source: Cell Death Dis. 2023 Jul 24;14(7):461. doi: 10.1038/s41419-023-05926-6 (PMC10366213; doi:10.1038/s41419-023-05926-6)
Supplement: Supplementary file 1 — Supplementary table 1. Human primers sequences. [file 41419_2023_5926_MOESM1_ESM.docx]

Supplement table 1. Human primers sequences.

| ID | Primer name | Direction | Primer seq | product | Tm | Gc% |
| --- | --- | --- | --- | --- | --- | --- |
| 1 | h_IHH_fwd | fwd | CTGGCCAATGTGACCGTAGT | 102 | 60.04 | 55 |
| 2 | h_IHH_rev | rev | GTGGGGATCATGGTTCAGCA |  | 60.03 | 55 |
| 3 | h_PTCH1_fwd | fwd | TGTGGCTGAGAGCGAAGTTT | 84 | 59.89 | 50 |
| 4 | h_PTCH1_rev | rev | CCACAACCAAGAACTTGCCG |  | 59.97 | 55 |
| 5 | h_SMO_fwd | fwd | CAGGGCACCGTATTCCTCTC | 175 | 59.89 | 60 |
| 6 | h_SMO_rev | rev | AAACGCAAAGAGGTTGGCAC |  | 59.9 | 50 |
| 7 | h_GLI1_fwd | fwd | CTCTTGCTTCCAGCTACCCC | 73 | 60.11 | 60 |
| 8 | h_GLI1_rev | rev | GCCCCTCACCTCCCTTCTAT |  | 60.4 | 60 |
| 9 | h_GLI2_fwd | fwd | TGCAACGTCCACCCACTTTA | 118 | 59.82 | 50 |
| 10 | h_GLI2_rev | rev | GGATACCAGGCATGCACACT |  | 60.11 | 55 |
| 11 | h_CDK1_fwd | fwd | TGCTTATGCAGGATTCCAGGT | 96 | 59.43 | 47.62 |
| 12 | h_CDK1_rev | rev | CCATGTACTGACCAGGAGGGA |  | 60.62 | 57.14 |
| 13 | h_Cyclin A2 _fwd | fwd | GCACTGGTGGTCTGTGTTCT | 144 | 60.18 | 55 |
| 14 | h_Cyclin A2_rev | rev | TGGATGCCAGTCTTACTCATAGC |  | 59.93 | 47.83 |
| 15 | h_Caspase3 _fwd | fwd | TGGTTTGAGCCTGAGCAGAG | 122 | 59.96 | 55 |
| 16 | h_Caspase3_rev | rev | TGGCAGCATCATCCACACAT |  | 60.03 | 50 |
| 17 | h_POU2F1 _fwd | fwd | ACCCAGCACTTGCATTAGTCT | 110 | 59.65 | 47.62 |
| 18 | h_POU2F1_rev | rev | GAAACAAACAAGAAGTTAAACGACA |  | 57.22 | 32 |
| 19 | h_SNAPC1 _fwd | fwd | GGACCCAAGTGATCGTGTGA | 133 | 59.68 | 55 |
| 20 | h_SNAPC1_rev | rev | GCTGAGGGCTTTATCTGGCT |  | 59.82 | 55 |
| 21 | h_POLR1B _fwd | fwd | CCTCGGAACCCAACTATCGG | 192 | 59.9 | 60 |
| 22 | h_POLR1B_rev | rev | AGACTTCCCGGCCATACTCT |  | 60.03 | 55 |
