# Supplementary material for: GANT-61 induces cell cycle resting and autophagy by down-regulating RNAP III signal pathway and tRNA-Gly-CCC synthesis to combate chondrosarcoma
Source: Cell Death Dis. 2023 Jul 24;14(7):461. doi: 10.1038/s41419-023-05926-6 (PMC10366213; doi:10.1038/s41419-023-05926-6)

Figure 3 full western blot

GLI1

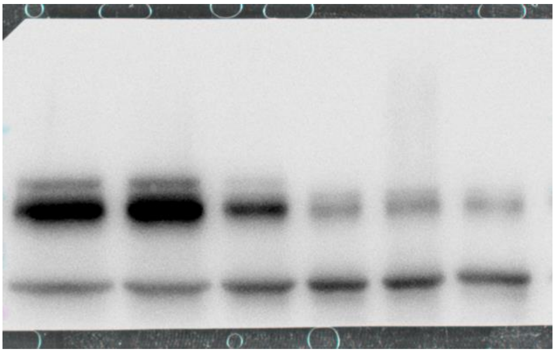

GLI2

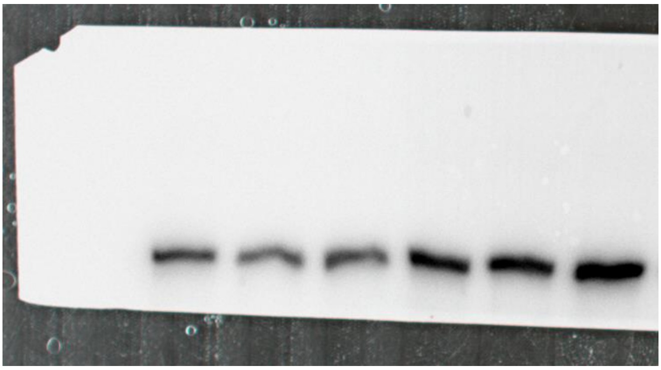

β-actin

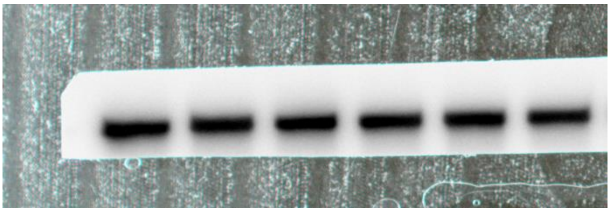

Figure 4 full western blot

CDK1

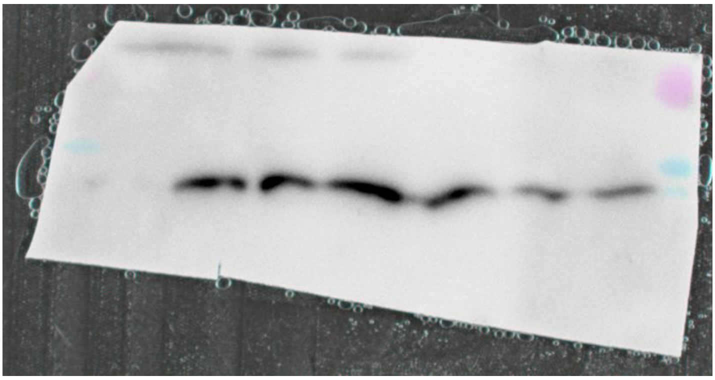

Cyclin A2

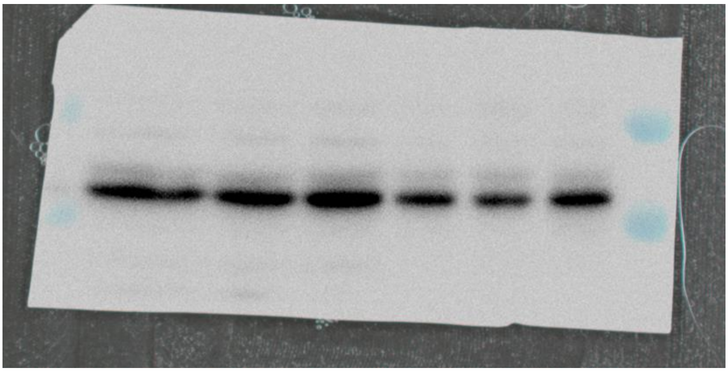

Cleaved-
Casepase 3

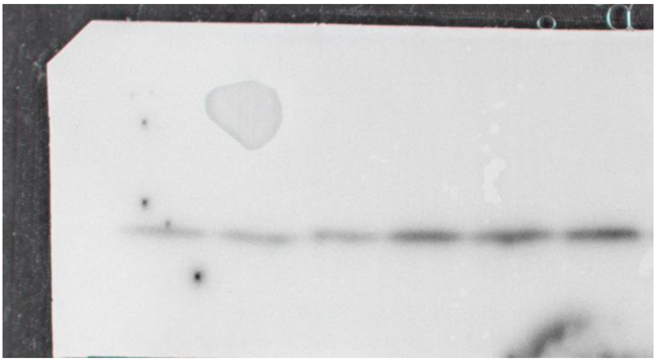

β-actin

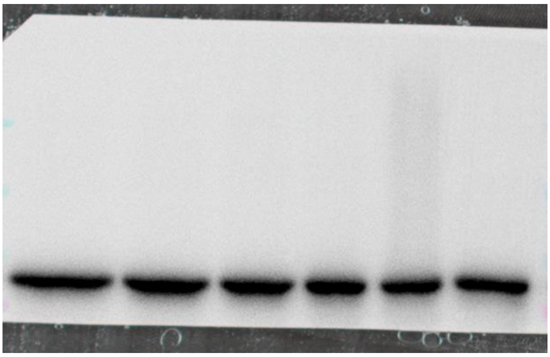

Figure 5 full western blot

P62

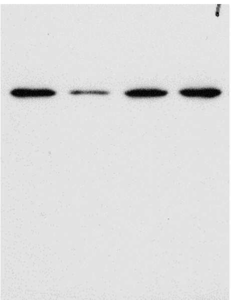

LC3B

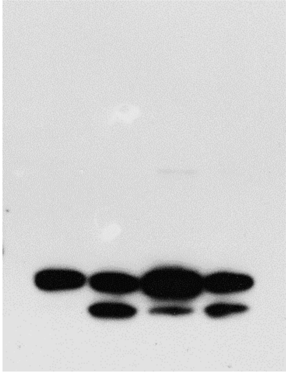

β-actin

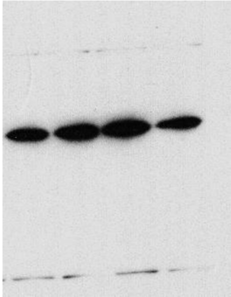

Supplement: Supplementary file 3 — Full western blot [file 41419_2023_5926_MOESM3_ESM.pdf]
